# Morphology and Molecules Reveal Unexpected Cryptic Diversity in the Enigmatic Genus *Sinobirma* Bryk, 1944 (Lepidoptera: Saturniidae)

**DOI:** 10.1371/journal.pone.0043920

**Published:** 2012-09-19

**Authors:** Rodolphe Rougerie, Stefan Naumann, Wolfgang A. Nässig

**Affiliations:** 1 Laboratoire d'Écologie, Université de Rouen, Mont Saint Aignan, France; 2 Independent Researcher, Berlin, Germany; 3 Entomologie II, Forschungsinstitut Senckenberg, Frankfurt am Main, Germany; Biodiversity Insitute of Ontario - University of Guelph, Canada

## Abstract

The wild silkmoth genus *Sinobirma* Bryk, 1944 is a poorly known monotypic taxon from the eastern end of the Himalaya Range. It was convincingly proposed to be closely related to some members of an exclusively Afro-tropical group of Saturniidae, but its biogeographical and evolutionary history remains enigmatic. After examining recently collected material from Tibet, northern India, and northeastern Myanmar, we realized that this unique species, *S. malaisei* Bryk, 1944 only known so far from a few specimens and from a very restricted area near the border between north-eastern Myanmar and the Yunnan province of China, may in fact belong to a group of closely related cryptic species. In this work, we combined morphological comparative study, DNA barcoding, and the sequences of a nuclear marker (D2 expansion segment of the 28S rRNA gene) to unequivocally delimit three distinct species in the genus *Sinobirma*, of which two are described as new to science: *S. myanmarensis* sp. n. and *S. bouyeri* sp. n. An informative DNA barcode sequence was obtained from the female holotype of *S. malaisei*—collected in 1934—ensuring the proper assignation of this name to the newly collected and studied specimens. Our findings represent another example of the potential of coupling traditional taxonomy and DNA barcoding for revealing and solving difficult cases of cryptic diversity. This approach is now being generalized to the world fauna of Saturniidae, with the participation of most of the taxonomists studying these moths.

## Introduction

The genus *Sinobirma* was described by Bryk [1] as a subgenus of the Indo-Australian genus *Opodiphthera* Wallengren, 1858. It comprises a single species, *Sinobirma malaisei* (Bryk, 1944), discovered in 1934 by the Swedish explorer and entomologist René Edmond Malaise during a very fruitful expedition at the Sino-Burmese border [1]. Forgotten for half a century, this moth was redescribed from Malaise's original material by Nässig & Oberprieler [2] and raised to genus level based on morphological differences in wing pattern, antennae and male genitalia structures. Most notably, these authors revealed the phylogenetic uniqueness of this genus and hypothesized that it is the first non-Afrotropical representative of the tribe Urotini Packard, 1902 (mentioned by Nässig & Oberprieler as Pseudapheliini Packard, 1914, but see Oberprieler [3] for the correct name, and an overview of the nomenclature in Naumann [4]) within the subfamily Saturniinae.

At the turn of the 21^st^ century, new material became finally available for study [5]–[7], bringing further knowledge about this peculiar species whose unique distribution and relationships within the family Saturniidae remain of enigmatic origin, possibly Gondwanan [2], [5]. In particular, the distinctly different phenotype of a few female specimens from Tibet encouraged us to look more closely at this material. Concomittently, the ongoing DNA barcoding campaign for saturniid moths (see Lepidoptera Barcode of Life at www.lepbarcoding.org; e.g. [8], [9]) offered the opportunity to also investigate genetic variation among the material available and proved decisive in sorting specimens of *Sinobirma malaisei* from various origins, as well as in understanding the significance of the subtle morphological variations preliminarily observed.

The present study proposes the description of two new species closely related to *Sinobirma malaisei*, based on morphology, DNA barcodes and the congruent evidence from a nuclear marker (28S rRNA). The type specimen of *S. malaisei* was also examined and sequenced to ensure the correct identity of our specimens.

## Materials and Methods

A detailed list of the material examined is given for each species in the taxonomic account below. All necessary permits were obtained for the described field studies from the Myanmarese Ministry of Travel and Tourism, and all relevant institutions and collections granted us permission to access and study the material used in this work. Genitalia preparation followed standard procedure using boiling 3% NaOH or 10% KOH solutions to digest internal tissues; after a careful cleansing and the removal of scales, the genitalia were examined, compared and described before being mounted onto microscope slides in Euparal.

### Genetic analyses

DNA was extracted from the legs of dried specimens in the collections of the authors, of colleagues, and in museum collections. A total of 17 specimens were processed (Table 1), including the holotype of *Sinobirma malaisei*. DNA barcodes (658 bp of the COI mitochondrial gene) were generated following the routine procedure at the Canadian Centre for DNA Barcoding (CCDB) as detailed in Vaglia et al. [10]. Because of its age, the holotype of *S. malaisei* was subsequently processed using alternative primer pairs developed by RR for archival material of sphingid moths and already known to be efficient in other families for specimens as old as 150 years [11]–[13]. These primer pairs target six slightly overlapping fragments of 90–120 bp along the barcode region of the COI gene.

**Table 1:** List of the samples used for the genetic analysis.

Taxon	Sex	Dep.	Country, region	Alt. (m)	SampleID	COI	28S
***Sinobirma malaisei*** ** HT**	F	NHRS	Myanmar, Kachin	2000	barcode SNB 1700	658[230n]/JN704367	
	F	MNHN	China, Yunnan	2080	BC-MNHN0016	658/JN704363	525/JN704392
	M	MNHN	China, Yunnan	2080	BC-MNHN0017	658/JN704364	
	M	MNHN	China, Yunnan	2080	BC-MNHN0018	609/JN704365	525/JN704393
	M	RCRR	China, Yunnan	2080	BC-Roug0001	658/JN704366	525/JN704394
	M	CSNB	China,Yunnan	1700	barcode SNB 618	658/JN704368	
	M	CSNB	China, Yunnan	1470	barcode SNB 1687	658/GU703010	525/JN704396
***S. myanmarensis*** ** HT**	M	MNHU	Myanmar, Kachin	1850	barcode SNB 617	658/JN704369	511/JN704397
**PT**	M	CSNB	Myanmar, Sagaing	1000	barcode SNB 613	645/JN704373	511/JN704401
**PT**	M	CSNB	Myanmar, Sagaing	1000	barcode SNB 614	658/JN704372	511/JN704400
**PT**	M	CSNB	Myanmar, Kachin	950	barcode SNB 615	658/JN704371	511/JN704399
**PT**	M	CSNB	Myanmar, Kachin	1850	barcode SNB 616	658/JN704370	511/JN704398
***S. bouyeri*** ** HT**	F	SMFL	China, Tibet	2000	BC-TB0165	658/JN704362	
**PT**	F	CTBO	China, Tibet	2000	BC-TB0166	658/JN704361	525/JN704391
**PT**	F	CTBO	China, Tibet	2000	BC-TB0167	658/JN704360	525/JN704390
**PT**	M	CSLL	India, Arunachal P.	2000	barcode SNB 1562	658/HM416785	
**PT**	M	CSNB	India, Arunachal P.	2000	barcode SNB 1688	649/GU703007	525/JN704395
***Maltagorea auricolor***	F	MNHN	Madagascar, Masoala	20	BC-MNHN0072	658/JN704359	

HT = holotype; PT = paratype; Dep. = depository collection (see text for abbreviations); F = female; M = male. SampleID codes are unique identifiers referring to individual records in the Barcode of Life Datasystems (BOLD, www.boldsystems.org); sequence length (bp) and GenBank accession numbers are given in separate columns for each gene.

Seeking for additional and independent evidence for the discrimination of the studied taxa, we amplified the D2 expansion segment of the ribosomal large subunit (28S) using the primer pair D2B and D3Ar [14]. The PCR cocktail was identical to the one used for COI, but a different thermocycling regime was employed: 1 cycle of 2 minutes at 94°C, 35 cycles of 30 seconds at 94°C, 30 seconds at 56°C, and 2 minutes at 72°C, with a final step of 2 minutes at 72°C.

DNA sequences, specimen data and images can be publicly accessed from BOLD, the Barcode of Life Datasystems [15] within dataset SINOB01. Sequences have also been deposited in GenBank (see Table 1 for accession numbers of the two genes). All sequences were aligned by eye; 28S rRNA sequences varied in length between 511 and 525 bp; the positioning of several indels along the sequences was straightforward for our limited set of taxa. COI barcode sequences presented no variation in length. The quantification of sequence divergences was performed using the Kimura 2-parameter (K2P) method in MEGA5 [16], [17] including all sites, with the pairwise deletion option and assuming both an homogeneous pattern of divergence among lineages and a uniform rate of substitutions among sites.

Phylogenetic analyses of DNA barcode sequences were carried out using the Madagascan species *Maltagorea auricolor* (Mabille, 1879) as an outgroup; the genus *Maltagorea* Bouyer, 1993 belongs to the tribe Urotini and was proposed as a possible sister taxon to *Sinobirma* on the basis of shared morphological characters [5]. The two genes were analysed separately and combined, using maximum parsimony (MP) and maximum likelihood (ML) methods. MP analyses were carried out in MEGA 5, including all sites and using CNI with search level 3 and 50 initial trees. The stability of each node was evaluated through a 500 bootstrap (BS) re-sampling. ML phylogenetic analyses were also performed using MEGA 5 with GTR and GTR+G+I models of evolution for 28S and COI DNA barcodes, respectively, as determined using jModelTest v.0.0.1 [18], [19]. The two genes were analysed simultaneously and un-partitioned in MEGA 5 with the GTR+G+I model. BS support was evaluated through 500 pseudo-replicates.

### Nomenclatural acts

The electronic version of this document does not represent a published work according to the International Code of Zoological Nomenclature (ICZN), and hence the nomenclatural acts contained in the electronic version are not available under that Code from the electronic edition. Therefore, a separate edition of this document was produced by a method that assures numerous identical and durable copies, and those copies were simultaneously obtainable (from the publication date noted on the first page of this article) for the purpose of providing a public and permanent scientific record, in accordance with Article 8.1 of the Code. The separate print-only edition is available on request from PLoS by sending a request to Public Library of Science, 1160 Battery Street, Suite 100, San Francisco, CA 94111, USA along with a check for $10 (to cover printing and postage) payable to ‘Public Library of Science’.

In addition, this published work and the nomenclatural acts it contains have been registered in ZooBank, the proposed online registration system for the ICZN. The ZooBank LSIDs (Life Science Identifiers) can be resolved and the associated information viewed through any standard web browser by appending the LSID to the prefix ‘http://zoobank.org/’. The LSID for this publication is: urn:lsid:zoobank.org:pub:352DF3F1-41DC-46D3-9E65-C3CE6FA84874. This article is deposited in the following digital archives: PubMedCentral and LOCKSS.

## Results

### Analyses of molecular data

First pass PCR amplification with the primer pair LepF1/LepR1, targeting a 658 bp fragment, was successful for all the specimens processed but for the old (ca. 75 y.o.) holotype of *Sinobirma malaisei*. This sample also failed to amplify when using the LepF1/MLepR1 and MLepF1/LepR1 primer pairs targeting shorter fragments of 307 and 407 bp, respectively. The approach using multiple minibarcodes produced, however, a partial DNA barcode missing 230 bases (between positions 230–360 and 431–528) for this specimen. We also obtained 28S rRNA sequences for 12 of the 16 samples processed for this gene (not attempted for the holotype of *S. malaisei*); the length of the sequences obtained was either 511 or 525 bp (Table 1; alignment given in Fig. S1).

**Figure 1:**
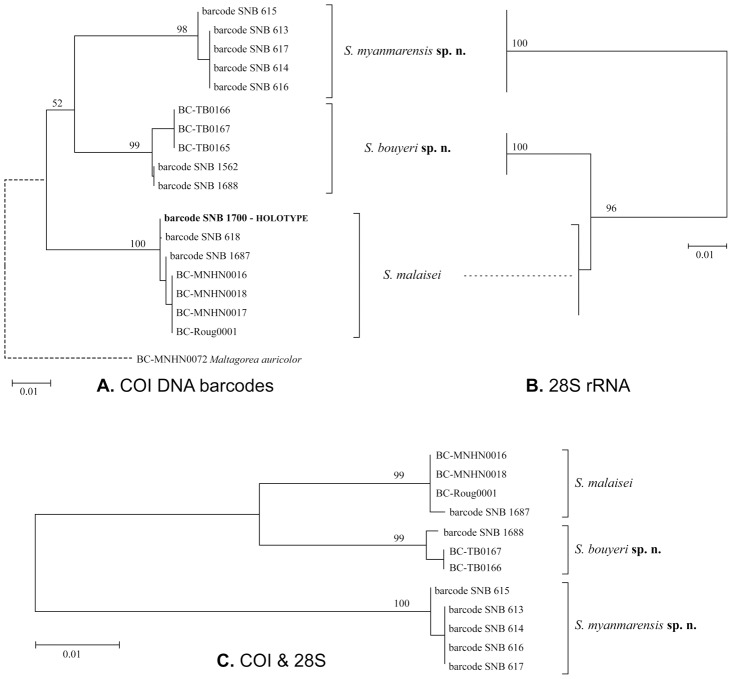
Phylogenetic analyses of sequence data. All trees shown are ML trees obtained from the analysis of (a) COI barcode sequences, (b) 28S rDNA sequences, and (c) the combination of both genes; values above branches are bootstrap supports for each node. Branch lengths represent the number of substitutions per site. Trees (b) and (c) are unrooted.

The analysis of DNA barcodes revealed three clearly separated groups of sequences (Fig. 1A), with minimal K2P distances between representatives of distinct clusters ranging between 4.4% and 5.4%, and maximal distances within each group between 0.3% and 0.6% (Table 2). The same three groups were supported as well by 28S rRNA sequences (Fig. 1B) which showed remarkably high levels of divergences between groups (Table 3), including several indels (Fig. S1).

**Table 2:** Percentage of divergence in DNA barcode sequences.

	*S. malaisei*	*S. bouyeri sp. n.*	*S. myanmarensis sp. n.*
***S. malaisei***	[0.3]		
***S. bouyeri*** ** sp. n.**	4.4	[0.6]	
***S. myanmarensis*** ** sp. n.**	5.4	4.6	[0.3]

Kimura 2-parameter (K2P) distances (%) for barcode DNA sequences of the three analyzed species in genus *Sinobirma*; minimal pairwise distances between species are given for each species pair; values in square brackets represent maximal intraspecific distances.

**Table 3:** Variations within sequences of 28S rRNA.

	Unique substitutions	Unique indels
***Sinobirma malaisei***	2	-
***S. bouyeri***	10 [1]	-
***S. malaisei + S. bouyeri***	43	5*

* indels length of 5, 17, 1, 2 and 3 bp in position 298, 306, 338, 372 and 448 respectively.

Sequence comparison of 28S rRNA sequences by reference to *Sinobirma myanmarensis*, the only species in three missing indels and tentatively considered as representing the ancestral condition (see text). Number within square brackets refers to substitutions located within indels.

Statistical support for the three groups was very high after analyzing the two genes separately or combined (Fig. 1, A–C) and independently of the phylogenetic method used (MP or ML), illustrating clearly the strong and highly congruent signal carried by these genes. The relationships between the three groups remain however unclear. When rooting the DNA barcode ML or MP trees with *Maltagorea auricolor*, the resulting topology (Fig. 1A) is only poorly supported (BS = 52). Our analyses of 28S sequences and of the combined datasets did not include an outgroup and the resulting trees (Fig. 1, B–C) are to be seen as unrooted (but see discussion below).

### Taxonomic account

**Figure 2:**
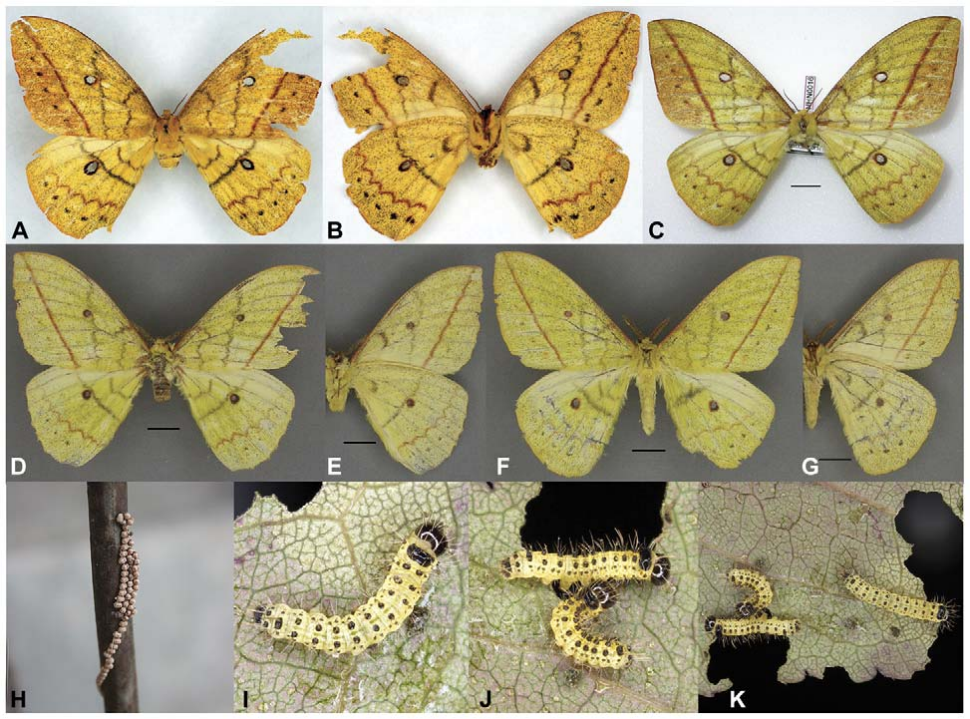
Specimens of *Sinobirma malaisei*; (A, C, D, F) = dorsal view, (B, E, G) = ventral view. Scale bars = 1 cm; specimens approximately to the same scale. (A–B): holotype ♀, Myanmar, Kachin State (S), Kambaiti; in NHRS. (C): ♀, China, Yunnan, Tongbinguan nature reserve; in MNHN. (D–E): ♂, China, Yunnan, Gaoligongshan; in CSNB. (F–G): ♂, China, Yunnan, Yingjiang, Xima; in CSNB. (H): ova, China, Yunnan. (I–K): 1^st^ larval instar, rearing attempt from the eggs in Fig. 1H.

**Figure 3:**
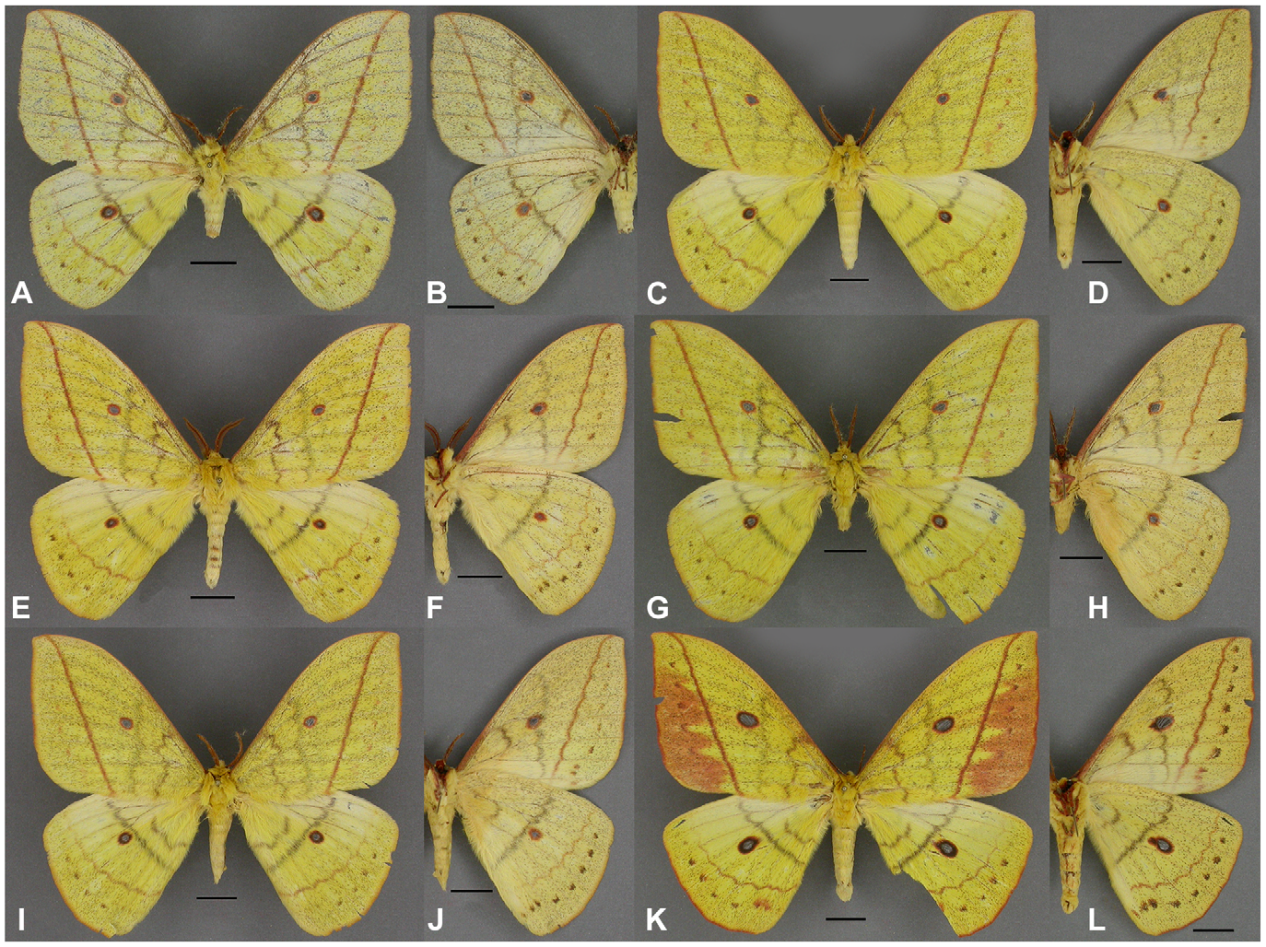
Specimens of *Sinobirma myanmarensis*; (A, C, E, G, I, K) = dorsal view, (B, D, F, H, J, L) = ventral view. Scale bars = 1 cm; specimens approximately to the same scale. (A–B): holotype (HT) ♂, Myanmar, Kachin State (N), Chudu Razi Range; in ZMHU. (C–D): paratype (PT) ♂, same data as HT; in CSNB. (E–F): PT ♂, Myanmar, Kachin State (N), E Putao; in CSNB. (G–L): PT ♂♂ (G–J) and ♀ (K–L), Myanmar, Sagaing State, E Ngalung Ga; in CSNB.

**Figure 4:**
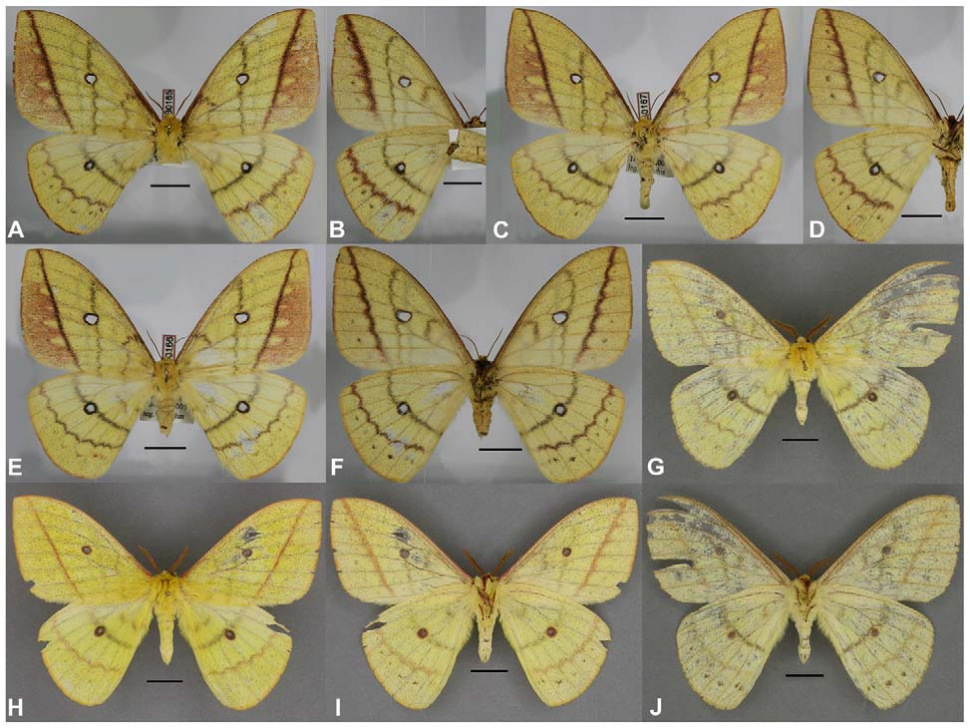
Specimens of *Sinobirma bouyeri*; (A, C, E, G, H) = dorsal view, (B, D, F, I, J) = ventral view. Scale bars = 1 cm; specimens approximately to the same scale. (A–B): holotype (HT) ♀, China, Tibet, Tomi; photo T. Bouyer; in SMFL. (C–F): paratype (PT) ♀♀, same data as HT; in CSNB & CTBO. (G–J): PT ♂♂, India, Arunachal Pradesh, near Rapum; in CSNB & CSLL.

**Figure 5:**
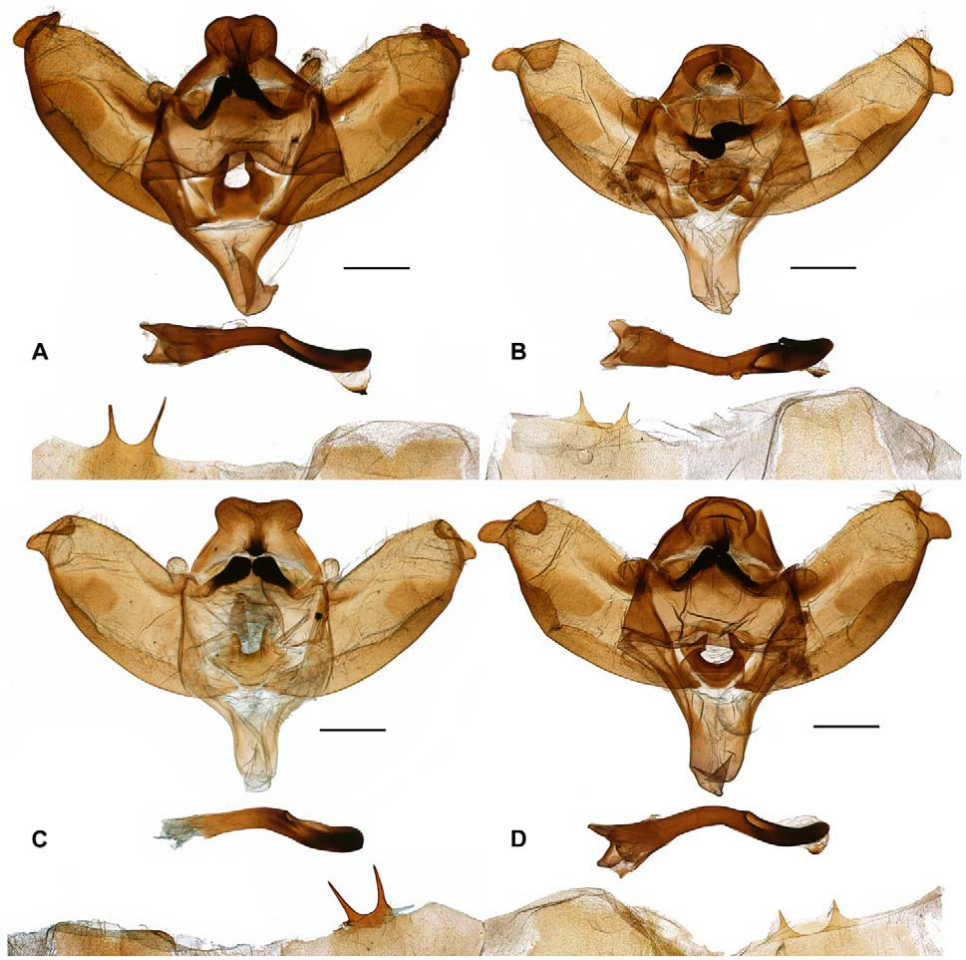
♂ genitalia of *Sinobirma* species; each panel represent a posterior view of the main genital structure at the top, the phallus immediately below, and the posterior end of 8^th^ sternite and tergite at the bottom. Scale bars = 1 mm. (A): *S. malaisei*. China, Yunnan, Gaoligongshan; no. 2024/09 Naumann. (B): *S. myanmarensis*. Myanmar, Kachin, Chudu Razi Range, no. 2022/09 Naumann. (C): *S. bouyeri*. India, Arunachal Pradesh, near Rapum, no. 2021/09 Naumann. (D): *S. myanmarensis*. Myanmar, Sagaing, E Ngalung Ga, no. 2023/09 Naumann.

**Figure 6:**
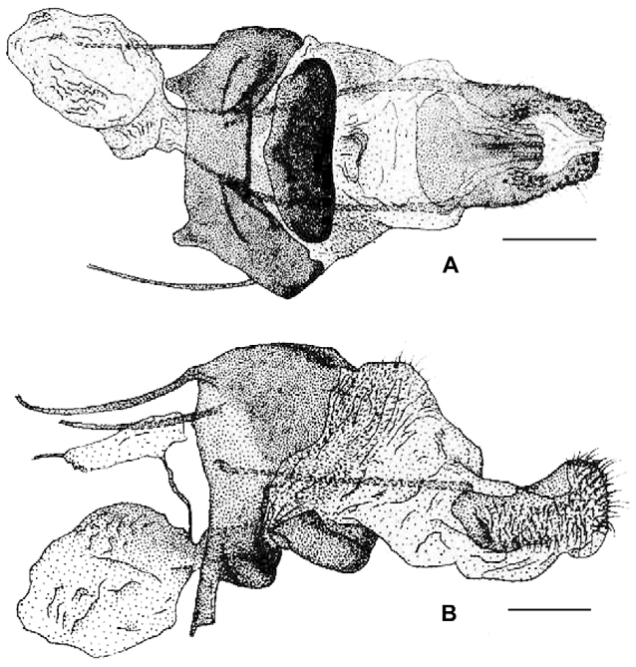
Female genitalia of *Sinobirma malaisei*, China, Yunnan, Tongbinguan nature reserve; (A) ventral view, (B) lateral view (drawings reproduced from [**5**] **).** Scale bars = 1 mm.

**Figure 7:**
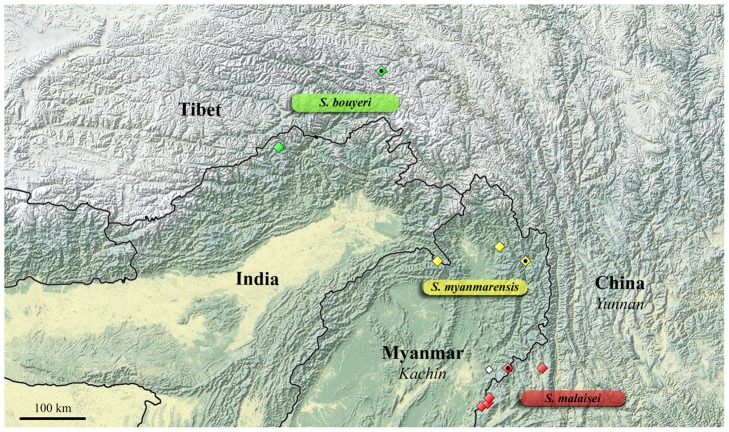
Map showing the known distribution of the species of *Sinobirma* in Asia. Symbols may represent more than one locality in close proximity; type localities are indicated by a black dot within the symbol. The locality cited in Vinciguerra & Racheli [6] for *S. malaisei* is represented by a white symbol.

These results, in conjunction with a thorough morphological comparison, confirm the existence among the material studied of three distinct species within the genus *Sinobirma*. In this section we propose a taxonomic account for the genus including (1) a re-description of the type-species of the genus, *S. malaisei*, whose identity was clearly established after sequencing of the DNA barcode of its holotype, and (2) the description of two new species. Adult specimens of both sexes, eggs and first instar caterpillars of *S. malaisei* are illustrated (Fig. 2), as well as male and female adults of the two newly described species (Fig. 3 for *S. myanmarensis* sp. n.; Fig. 4 for *S. bouyeri* sp. n.) and the genital structures of males for the three species (Fig. 5) and of the female for *S. malaisei* only (Fig. 6). A map summarizing known distribution of the three species is also provided (Fig. 7).

### *Sinobirma* Bryk, 1944

Type species: *Opodiphthera (Sinobirma) malaisei* Bryk, 1944, by original designation.

Note: In the online database (http://www2.nrm.se/en/lep_nrm/lepidopterakopia.html) of the Naturhistoriska Riksmuseet (Stockholm, Sweden) the genus and species *Sinobirma malaisei* Bryk (and other taxa described in the same publication) are cited with the date “1943", probably based on the submission date printed on the first page of the contribution. However, in the original publication [1], there is on p. 55 an imprint date of “31 January 1944", and in the list of contents of volume 35 of *Arkiv för Zoologi*, it is announced that “Häftet 2" containing Bryk's contribution (beside others and as no. 8) was published on “4 April 1944", and so the publication date 1944 is clear.

Bryk [1], despite questioning himself the discovery of a member of a typically Australian genus on the fringe of the Palaearctic faunal region, still placed his new species in the genus *Opodiphthera* within which he erected a new subgenus, *Sinobirma*, for the only known species at the time. This classification was only based on superficial similarity of the taxa involved. The type species of *Opodiphthera* is the Australian *O. varicolor* Wallengren, 1858, which was figured as well in Bryk's work. This name is a junior subjective synonym of *O. astrophela* (Walker, 1855) [20]. Since its description, *Sinobirma* was not mentioned again in literature until Nässig & Oberprieler [2] studied the original material of Bryk and raised the subgenus to generic level, also proposing a relationship between *Sinobirma* and a group of African Urotini genera closely related to *Tagoropsis* C. & R. Felder, 1874. These unexpected affinities of *Sinobirma* make its evolutionary and biogeographical history enigmatic and of particular interest, this taxon being seen as a possible Gondwanan relict [2], [5].

The genus was not mentioned in Chinese literature on Saturniidae so far (review, e.g., in Zhu & Wang [21]). It is only during the past decade that more material became available and was cited in a few publications [5]–[7]. Specimens are rare in collections, possibly because of their short period of flight during the rainy season and in mountainous areas which are difficult to access. Representatives of the genus are only known thus far from medium (950 m) to relatively high (2200 m) elevation localities at the eastern edge of the Himalaya Mountains (Fig. 7).

### Sinobirma malaisei (Bryk, 1944)

#### Holotype (by original designation) ♀

N.E. Burma [ = Myanmar, Kachin State, Chinese borderline], Kambaiti, 2000 m, 12–17.vi. 1934, R. Malaise leg., genitalia no. 53 Bouyer = 11083 NHRS (in Naturhistoriska Riksmuseet, Stockholm, Sweden = NHRS); BOLD sampleID: barcode SNB1700 (Fig. 2, A–B).

#### Paratypes (3 ♂♂)

N.E. Burma [ = Myanmar, Kachin State, near Chinese borderline], Kambaiti, 2000 m, 9. & 17. vi. 1934, R. Malaise leg., genitalia no. 398/86 Nässig = 7176 NHRS (NHRS).

#### Further material examined

1 ♀, same locality as holotype, vi. 1934 (Staatliches Museum für Naturkunde, Karlsruhe, Germany = SMNK). 1 ♂, same locality as holotype, vi. 1934, genitalia no. 298/86 Nässig (Museum Alexander Koenig, Bonn, Germany = MAKB). 4 ♂♂, 2 ♀♀, PR China, Yunnan, Tongbinguan nature reserve, Road from Xima to Myanmar border, km. 12, 24°49′N, 97°44′E, 2080 m, 12.–13.vi. 2001, leg. R. Rougerie, A. Mantilleri, Tian Mingyi & T. Deuve, genitalia RR085, RR118, RR224 in Muséum National d'Histoire naturelle, Paris, France = MNHN, BOLD sampleID: BC-MNHN0016 (Fig. 2C), BC-MNHN0017, BC-MNHN0018, BC-Roug0001 (MNHN, Research Collection of Rodolphe Rougerie, Rouen, France = RCRR). 1 ♂, China, West Yunnan, Gaoligongshan Mts., Da-Tang, a village 80 km in the north of Teng-chong county [*sic*], ca. 1700–2000 m, 17.–22. v. 2005, leg. Huang Hao (Collection Wolfgang A. Nässig = CWAN, now hosted in Senckenberg-Museum, Lepidoptera collection, Frankfurt am Main, Germany = SMFL). 1 ♂, same data (Fig. 2, D–E), genitalia no. 2024/09 Naumann, BOLD sampleID: barcode SNB 0618 (Collection Stefan Naumann, Berlin, Germany = CSNB). 7 ♂♂, PR China, Yunnan, Yingjiang County, Xima town, 1470 m, 27.–29.V.2009, leg. Weiwei Zhang (Collection Swen Löffler, Lichtenstein, Germany = CSLL). 1 ♂, same data (Fig. 2, F–G), BOLD sampleID: barcode SNB 1687 (CSNB).

The additional ♂ specimen from “Binguanshan, 2500 m, near Myitkyina" (Fig. 7 – white symbol) cited by Vinciguerra & Racheli [6] most likely also belongs to *S. malaisei*, although we note that we have not seen and studied this specimen. This mountain locality lies somewhere east of the city of Myitkyina, which is situated in the lowlands of the Irrawaddy river system, probably along the road to China via Kambaiti, and quite close to the type locality of *S. malaisei*.

#### Etymology

*S. malaisei* was named after the collector of the type series, René Malaise, who collected Lepidoptera on the Swedish expedition to Burma ( = Myanmar) and British India in 1934.

#### Distribution

The species is known from a few localities in the border area between Southern Kachin State in Myanmar and the Yunnan province in China (see Fig. 7), at altitudes between ca. 1700 and 2500 m.

#### Description

**♂** (Fig. 2, D–G): Forewing length, measured in a straight line from basis to apex, 45–51 mm. Ground colour is a pale yellow, interspersed with lots of greyish scales. Antennae bipectinate, pectination much reduced on the apical 4 or 5 joints; they are rusty-brown in fresh specimens [5], but later dry to some sort of olive-ochreous colour, with 35–36 segments, and have a maximum length of ca. 10 mm, longest rami are 1.3 mm long. Head, thorax and abdomen in ground colour, only ventral parts of the frons covered with purple hair. Forelegs have a purple medial part, and all legs purple tarsi, otherwise in ground colour. Forewing rounded, apex almost rectangular; on the dorsal side only the following pattern elements appear on the otherwise homogenous wings: costa with a purple shadow in the basal third. The forewing antemedian band grey, curved. The median band grey, almost straight or little dentate, situated proximal to the ocellus or touching it on proximal side. Forewing postmedian band reddish brown, straight. In the postmedian area a row of small dark grey dots. Forewing ocellus round, circled reddish brown on proximal side, black on distal side, 2.5–3.1 mm in diameter. Hindwing antemedian and median band dark grey, the median band touches the ocellus medially in most cases. Hindwing ocellus round, narrowly bordered reddish brown on proximal and broadly black on distal side, 2.9–3.5 mm in diameter. Postmedian band reddish brown, dentate, followed again by a row of dark grey dots. Marginal fringes of both fore- and hindwing yellow. Pattern on ventral side similar, colours are somewhat paler.

**♀** (Fig. 2, A–C): Females are very similar to males, with few differences: the ground colour is a little darker, the colouration of the antemedian and the median band is somewhat darker, more blackish, and the forewing postmedian area is more or less suffused with orange scales and darker black dots. On ventral side the forewing median line is in a different position than on the dorsal side, running posterior to the ocellus. The female shows further differences due to general sexual dimorphism: the wings are broader, slightly more rounded though still with an apex almost rectangular; the antennae are bipectinate with shorter rami, and the body is heavier. Forewing length of the holotype 51.6 mm.

#### ♂ genitalia (Fig. 5A)

Male genitalia structures were already described and illustrated by Nässig & Oberprieler [2] and Rougerie [5]; we here give again a short description as a reference for the comparative study with the two species described below. Uncus with two rounded dorsal processes with a slight dorsal indentation, ventrally slightly excavated. There is a disto-ventral hook-like tip. The median processes of the gnathos are not fused in the centre, there are separate right and left arms of the gnathos. However, there seems to be a certain amount of intraspecific variability of this structure in the entire genus. The two knob-like protuberances at the dorsal basis of the valves are not connected to the valves, but are lateral ends of the gnathos. Valves with a crown-like rounded apical process. Phallus with vesica emerging left lateral, the vesica has an apical sclerite with short spines emerging. The 8th abdominal sternite with two long slender processes, closely emerging from a mutual plate; the 8th tergite less sclerotised, sclerotization broad with acute lateral tips, median indentation narrow and deep.

#### ♀ genitalia (Fig. 6, A–B)

Ovipositor is formed by a pair of fleshy and setose papillae anales; posterior apophyses about one quarter longer than the anterior apophyses. Membranous area between papillae is slightly sclerotised with longitudinal folds; sterigma formed of a strongly sclerotised anterior part continuous with tergum A8, and of a large sclerotised and thickened lamella postvaginalis. Ostium bursae large, situated anterior to the lamella postvaginalis. Tergum A8 is divided by a membranous zone enlarged anteriorly. The ductus bursae is short, only weakly sclerotised dorsally; corpus bursae small with many wrinkles and no signum. The ductus seminalis enters on dorso-lateral right side of posterior part of the corpus bursae; spermatheca is large, nearly as long as the whole genital apparatus; internal and external canals short and very thin, converging towards a thick and long receptacular canal dividing into an ellipsoid lagena and a long utriculus approximately equal in length to that of lagena.

#### Preimaginal instars (Fig. 2, H–K)

The early stages of *S. malaisei* remain unknown, but for a few images of a failed rearing attempt by our colleague Steve Kohll (Luxembourg). Among several plants offered, only leaves of the ornamental *Prunus cerasifera* ‘var. Atropurpurea’ (Rosaceae) were accepted, but the larvae died soon after first moult. The eggs are ovoid, of whitish colour and covered with brown secretion to glue them on the substrate. They are deposited in long parallel rows, partly overlapping in three or four contiguous rows. Unfortunately, no ova of the rearing attempt were preserved, precluding measurements and examination of ultrastructure characters. First instar larvae live gregarious, have a pale yellow ground colour, a shiny black head, and black markings highlighting primary setae or groups of setae (tubercle-like scoli); the dorsal shield on thorax is large, black; dorsal scoli are separated along the body but for the 8^th^ abdominal segment where their black bases (not the scoli themselves) are fused; anal legs with black lateral plates and a black dorsal plate. All tubercles and head capsule are covered with rather short yellowish bristles. The larvae look similar in shape, but clearly different in colour and other details to those published and illustrated by Bouyer et al. [22] for the related *Pseudantheraea discrepans* (Butler, 1878).

#### Ecology and ethology

*S. malaisei* was reported to fly in a subtropical evergreen-broadleaved forest consisting of low and medium-sized trees, including numerous *Castanopsis* (Fagaceae) trees [5]. This region receives monsoonal precipitations and has mild climate. Observed flight period was about 9 PM for females and between 11:30 PM and midnight for males (local time). The village of Kambaiti (Myanmar, Kachin state), type locality of *S. malaisei*, was visited in 2010 by SN; although no collecting could be carried out in this area, the surrounding mountains appeared largely forested and favorable to the maintained presence of the moth (Fig. S2).

### *Sinobirma myanmarensis* Naumann, Nässig & Rougerie sp. n

urn:lsid:zoobank.org:act:EC7A752B-8A16-4CF4-A735-F2861EF074BE

#### Holotype ♂

Myanmar (NE), Kachin State, Chudu Razi Hills, 30 miles E Kawnlangphu, 29. v. 2007, leg. local collector, genitalia no. 2022/09 Naumann, BOLD SampleID: barcode SNB 0617 (CSNB), to be deposited in Museum für Naturkunde der Humboldt-Universität zu Berlin, Germany = MNHU (Fig. 3, A–B).

#### Paratypes (in total 22 ♂♂, 1 ♀)

5 ♂♂, same locality as holotype, 24. v., 1. vi., 2×2. vi. (Fig. 3, C–D), 20. vi. 2007, BOLD SampleID: barcode SNB 0616 (CSNB); 2 ♂♂, same locality, 1. & 2. vi. 2007 (CSLL); 1 ♂, same locality, 1. vi. 2007 (CWAN); 1 ♂, same locality, 3. vi. 2007 (Collection Thierry Bouyer, Chênée, Belgium = CTBO); 1 ♂, N. Myanmar (Burma) [Kachin State], Nan Thi, 50 km E Putao, 950 m, 21°21′N, 97°55′E, 11.–16. v. 1998, BOLD SampleID: barcode SNB 0615 (CSNB) (Fig. 3, E–F); 7 ♂♂, 1 ♀, Myanmar, Sagaing State (N), E Ngalung Ga, SSE Kumki (India), Tarung Hka river fork, 1 km E Hkasi village, 1000 m, 27°7.875′N, 96°53.105′E, 30. v., 10. vi., 6× [incl. ♀, Fig. 3, K–L] 11. vi. 2008, leg. local collector, genitalia ♂ no. 2023/09 and ♀ no. 2255/11 Naumann, BOLD SampleID: barcode SNB 0613 (Fig. 3, I–J) & 0614 (Fig. 3, G–H) (CSNB); 2 ♂♂, same locality, 30. v. & 11. vi. 2008 (CSLL); 1♂, same locality, 11. vi. 2008 (CTBO); 1♂, same locality, 11. vi. 2008 (coll. Y. Kishida, Tokyo, Japan); 1♂, same locality, 11. vi. 2008 (coll. T. Klemetti, Imatra, Finland).

#### Etymology

Named after the origin of the type material.

#### Distribution

Known from several localities in Myanmar: N. Kachin State and Sagaing State (see Fig. 7) at about 1000 m of altitude.

#### Description

**♂** (Fig. 3, A–J): Forewing length 48–55 mm (holotype: 48 mm). Ground colour similar to that of *S. malaisei*. Antennae bipectinate, pectination much reduced on the apical 4 or 5 joints; they are also of olive-ochreous colour (probably as well an alteration after the specimen dries out in collection) with 35–38 segments, and have a maximum length of ca. 11 mm, longest rami are 1.4 mm long. Head, thorax and abdomen as in *S. malaisei*, with same colouration of frons and legs. The forewing larger, more widened toward the anal margin, the apex almost rectangular, with the following consistent differences from *S. malaisei* in dorsal wing pattern elements: costa almost completely in ground colour, the forewing antemedian and median bands grey, more separated from each other than in *S. malaisei*, the median band distinctly proximal to the discal ocellus, rarely touching it. The postmedian band reddish brown and straight as in *S. malaisei*, but always distinctively bent toward the apex, not reaching the tip of the wing though. In the postmedian area a row of small reddish, more or less clearly marked dots. Forewing ocellus slightly ovoid, completely circled in reddish brown, 3.1–4.1 mm in diameter. Hindwing antemedian and median band dark grey, the median band is proximal to the discal ocellus, touching it in some specimens. Hindwing ocellus almost round, circled black internally and reddish brown externally, 3.1–3.9 mm in diameter. Postmedian band reddish brown, less dentate than in *S. malaisei*, wavy, followed again by a row of dark grey dots. Marginal fringes of both fore- and hindwings orange to reddish. Pattern on ventral side similar, colours are somewhat lighter; the median line of the forewing is more distal in position that on the dorsal side, running beyond or marginal to the ocellus.

**♀** (Fig. 3, K–L): As in *S. malaisei*, the female is very similar to the males, with few differences: the ground colour on dorsal side is slightly darker yellow than in males; colouration of antemedian and median band is also somewhat darker, the median band even a little brownish, the forewing postmedian area is orange but for its apical part and contains a row of dark reddish brown dots. As in the males, the straight postmedian line is distinctively bent apically towards the apex. On ventral side the forewing median line is in a more distal position than on the dorsal side; it runs through the discal and is very undulated. The female shows the standard differences due to sexual dimorphism: wings are broader, more rounded, yet with an almost rectangular apex; the latter very slightly pointed. The antennae bipectinate with shorter rami than males; the body is heavier. Forewing length 55 mm, forewing ocellus ovoid, 5.9 mm in diameter, that of the hindwing 5.5 mm. The antennae are ca. 10 mm long and have 33 segments.

#### ♂ genitalia (Fig. 5, B, D)

Uncus as in *S. malaisei*, with two rounded dorsal processes with a slight dorsal indentation, and a disto-ventral hook-like tip. As in *S. malaisei*, the median processes of the gnathos are not fused in the centre, there are separate right and left arms of the gnathos, also with a certain amount of intraspecific variability of this structure. The two knob-like protuberances at the dorsal basis of the valves are not connected with this structure, but are lateral ends of the gnathos. Valves with a well developed harpe and two rounded apical processes. Phallus with vesica emerging left-lateral, the vesica has an apical sclerite with short spines emerging. The 8th sternite with two shorter, quite acute processes, originating widely separated and without a mutual prolongation of the sternite at their base. The sclerotization of the 8th tergite slightly narrower in shape than in *S. malaisei*, with lateral tips narrower and more rounded, the median indentation wider and more shallow.

#### ♀ genitalia

The only dissection of a ♀ came from a specimen with dense fungi growth in the abdomen; the structures are distorted, partly destroyed and cannot be well separated from the fungi. It is not illustrated here but was found very similar to *S. malaisei*.

#### Biology

The preimaginal instars and ecology of the species are unknown.

#### Remarks

This new species occurs in the same mountain range than *S. malaisei*, east of the Nmai Hka fork of the Irrawaddy system. There is however a large gap between the distributions of the two species (Fig. 7); this area was prospected by SN during two expeditions in places between Kambaiti and the Chudu Razi Range in northeastern Myanmar, but no specimen of *Sinobirma* was collected. Further collecting efforts in this poorly known area will be necessary to assess if the ranges of *S. malaisei* and *S. myanmarensis* are actually disjunct or if the two species coexist in some places. *S. myanmarensis* was cited as *S. malaisei* in [5]–[7] [partim].

### *Sinobirma bouyeri* Naumann, Nässig & Rougerie sp. n

urn:lsid:zoobank.org:act:53E34B9B-70F5-4648-9C3C-B1598B09A71E

#### Holotype ♀

China, E. Tibet, Tomi, 2000 m, 10.–12. vi. 2005, leg. V. Paulus, BOLD SampleID: BC-TB0165, genitalia no. 2056/08 Nässig (ex CTBO), deposited in SMFL (Fig. 4, A–B).

#### Paratypes (7 ♂♂, 2 ♀♀)

1 ♀, same data as holotype, BOLD SampleID: BC-TB0166 (CTBO) (Fig. 4, E–F); 1 ♀, same data as holotype, BOLD SampleID: BC-TB0167 (CSNB) (Fig. 4, C–D). 6 ♂♂, India, Arunachal Pradesh, Dist. Along, near Rapum, 2000 m, 28.53176°N, 94.24941°E, 9.–11. v. 2009, leg. G. Bretschneider (1 ♂ Fig. 4, H–I), 1 ♂ with genitalia no. 2021/09 Naumann, BOLD SampleID: barcode SNB 1562 (all CSLL); 1 ♂, same data, BOLD SampleID: barcode SNB 1688 (Fig. 4, G, J) (CSNB).

#### Etymology

This species is dedicated to Thierry Bouyer, who was the first to point at the peculiarity of the Tibetan specimens.

#### Distribution

Known from the type locality in Tibet, and from India, Arunachal Pradesh (Fig. 7), at 2000 m elevation. These two localities are almost 300 km apart and separated by high mountains and deep valleys. We note that the type locality of this species, reading “Tomi" on the label, was somewhat enigmatic. We could not locate “Tomi" in any map or gazetteer at first; however, a slightly more complete label of a specimen of *Saturnia tibetanna* Naumann & Nässig, 2010 in the CSNB, collected in 1996 at the same locality and by the same person (but with elevation data 2200 m), helped to locate this site. This label (see figure 50a in Naumann & Nässig [23]) reads “Tomi (Tangmai)", and further research revealed it to be a truckstop with restauration at 30°8′N, 96°5′E in East Tibet.

#### Description: ♂ (Fig. 4, G–J)

Forewing length 39–45 mm. Ground colour is a paler yellow when compared to the two species mentioned above, interspersed with lots of greyish scales. Antennae bipectinate, pectination much reduced on the apical 4 or 5 antennal segments; these are of olive-ochreous colour (supposedly also an altered colour as evidenced in *S. malaisei*), with 34–35 segments, and have a length of ca. 10 mm, longest rami are 1.4 mm long. Head, thorax and abdomen with same colouration of frons and legs as in the other species. The forewing smaller, rounded, even so in the apical area, with the following pattern elements on the otherwise homogenous wings on dorsal side, which differ constantly in detail from those of *S. malaisei* and *S. myanmarensis*: costa with a purple hue in the basal part; the forewing antemedian and median bands much reduced, greyish, only weakly visible; the almost straight median band is situated distantly proximal to the ocellus. Also, the forewing postmedian band is reddish brown, straight, ending at the costa further away from the apex than in *S. malaisei*. In the postmedian area a row of small dark grey dots visible in some of the specimens only. Forewing ocellus round, circled reddish brown to black with some individual variation, 2.2–3.0 mm in diameter. Hindwing antemedian and median band dark grey, the well-marked median band goes straight through the ocellus. Hindwing ocellus round, circled black, 2.8–3.2 mm in diameter. Postmedian band reddish brown, undulated, followed by a row of dark grey dots. Marginal fringes of both fore- and hindwing yellowish. Pattern on ventral side similar, colours are somewhat lighter, the median line of the forewing situated posterior to the ocellus or, in some specimens, completely missing.

**♀** (Fig. 4, A–F): The females are very similar to the males, with a few differences: the forewing costa is reddish brown in its proximal half; the colouration of antemedian, median and postmedian bands is much darker and more intense than in the two previous species. Forewing postmedian area orange but for its apical part, as in the single known female of *S. myanmarensis*. As in all other species, the position of the forewing median line on the ventral side differs from that of the dorsal side, running in a more distal position, in contact with or beyond the forewing ocellus. The postmedian lines of both fore- and hindwings are more heavily marked ventrally, with some parts of the pattern extending into the postmedian areas, especially along the veins, thus making those lines appearing somewhat undulated. The females present the usual differences related to sexual dimorphism: wings are somewhat more rounded and broader, the antennae have shorter rami, and the body is heavier. Forewing length of holotype 45 mm.

#### ♂ genitalia (Fig. 5C)

Uncus as in *S. malaisei* and *S. myanmarensis*, with two rounded dorsal processes which are more deeply indented medially, and with a more pronounced disto-ventral hook-like tip. As in the other two species, the median processes of the gnathos are not fused in the centre, and there are separate right and left arms of the gnathos. The two knob-like protuberances at the dorsal basis of the valves again are lateral ends of the gnathos and are not connected with the valves. Valves with two rounded apical processes. Phallus with vesica emerging left-lateral, the vesica has a small apical sclerite. The 8th sternite with two long, slender processes on a mutual plate; the sternite is distinctly shorter than in *S. malaisei*. The shape of the sclerotized part of the 8^th^ tergite is trapezoid and broader, the lateral tips rounded, and the central indentation very shallow.

#### ♀ genitalia

Similar to *S. malaisei*, but not illustrated and compared, as the holotype genitalia are damaged and incomplete (decayed by bacteria).

The preimaginal instars and ecology of the species are unknown.

## Discussion

It is remarkable that the material recently made available for the genus *Sinobirma*, very rarely collected moths, proved to encompass three distinct species in the Indo-Himalayan region. While this material had first been considered to match the only existing species *Sinobirma malaisei*, preliminarily observed subtle differences (in particular for these female specimens from Tibet) were rapidly confirmed by genetic data (DNA barcodes at first) and led to the present more comprehensive study integrating morphology, mitochondrial DNA and nuclear DNA data. This study not only confirmed the specific status of the moths flying in Tibet, *Sinobirma bouyeri*, but also revealed the existence of a third species, *Sinobirma myanmarensis*, distributed in the northern Kachin and Sagaing states of Myanmar. The three species of *Sinobirma* can be determined externally by their combination of morphological characters (see Table 4); in particular, while *S. malaisei* and *S. myanmarensis* look superficially very similar, they exhibit the largest differences in male genitalia. *S. bouyeri*, which superficially looks rather distinct, has very similar genitalia to *S. myanmarensis*, but harbours distinct sclerotized parts of the 8th abdominal sternite. DNA barcodes of the three species are very distinctive (Fig. 1A, Table 1) and the successful sequencing of this marker for the holotype of *S. malaisei* has established beyond doubt the identity of the material from China (Yunnan); the latter had been collected in Chinese localities very close to the border with Myanmar where the type-locality of *S. malaisei* lies (Kambaiti, Kachin state [5], Figs. 7, S2), but specimens of *S. myanmarensis*, collected further north in the same state, might have been conspecific with the type.

**Table 4:** Summary of the material examined and of diagnostic morphological characters between the three *Sinobirma* species studied.

	*S. malaisei*		*S. myanmarensis*	*S. bouyeri*	
	Myanmar, Kambaiti (type loc.): 4 ♂♂, 2 ♀♀)	China, Yunnan: 15 ♂♂, 2 ♀♀	Myanmar, N. Kachin & Sagaing: 20 ♂♂, 1 ♀	China, Tibet: 3 ♀♀	India, Arunachal Pradesh: 7 ♂♂
**Wings, dorsal surface (Fw = forewing; Hw = hindwing)**					
**Fw antemedian band**	grey	grey	grey	grey	only weakly marked, grey
**Fw median band**	grey, touching discal ocellus proximally	grey, touching discal ocellus proximally in most but not all specimens	grey, proximal to discal ocellus, rarely touching it	black, running through ocellus centre or touching it proximally	weakly indicated, grey, proximal and distant to ocellus
**Fw postmedian band**	reddish brown, straight and ending near the apex	reddish brown, straight and ending near the apex	reddish brown, straight, bent towards apex	dark reddish brown, broad, straight and ending near the apex like in *S. malaisei*	reddish brown, straight, ending further away from the apex than in *S. malaisei*
**Hw antemedian band**	grey	grey	grey	grey, weakly marked	grey
**Hw median band**	grey, touching discal ocellus proximally	grey, touching discal ocellus proximally	grey, proximal to discal ocellus, sometimes touching it	dark grey, straight through ocellus	grey, straight through ocellus
**Hw postmedian band**	reddish brown, zig-zagged	reddish brown, zig-zagged	reddish brown, wavy	dark reddish brown, wavy	reddish brown, wavy
**Fw ocellus**	♂♂ & ♀♀: circled reddish brown proximally, distally black	♂♂ & ♀♀: circled reddish brown proximally, distally black	♂♂: circled reddish brown, ♀: internal ring black, external ring red	♀♀: circled black	♂♂: circled dark reddish brown to black, slight variation
**Hw ocellus**	♂♂ & ♀♀: circled black, with reddish proximal blur	♂♂: circled in thick black distally, narrow reddish brown proximally; ♀♀: reddish proximal part thicker	♂♂: circled black internally, reddish brown externally ♀: thick black ring internally, reddish brown ring externally	♀♀: thick black circle	♂♂: thick black circle
**Outer marginal fringes of wings**	yellowish	yellowish	orangy to reddish	dark reddish brown	yellowish
**Other remarkable observations**	♀♀: Fw apex almost rectangular; reddish colour in Fw postmedian field less intense than in other species	♀♀: Fw apex almost rectangular , reddish colour in Fw postmedian field less intense than in other species	♀: with slightly acute fw apex; reddish colour in Fw postmedian field more intense	♀♀: Fw apex almost rectangular; wings more rounded; reddish colour in Fw postmedian field more intense	♂♂: wingshape more rounded
**♂ Genitalia**					
**8th sternite**	with two long slender processes closely originating from a mutual plate		with two distant, short and quite acute processes, originating from the posterior margin of the sternite	(♀♀ only)	distinctly shorter than in *S. malaisei*, with two long, slender processes on a mutual plate
**8th tergite**	broader than in other species, with posterior angles more acute and a narrow and deep median indentation in its posterior margin		narrower, posterior angles more rounded, median indentation broad and shallow	(♀♀ only)	broad, trapezoid, with posterior angles rounded, median indentation very small
**Uncus**	dorsal processes rounded, a slight central indentation, ear-like, slightly excavated ventrally; hook-like tip disto-ventrally		as in *S. malaisei*	(♀♀ only)	dorsal processes slightly more deeply indentated; disto-ventral hook more pronounced
**Valves**	two closely contiguous small apical lobes		two apical lobes more prominent and separated (esp. ventral lobe)	(♀♀ only)	two apical lobes intermediate between the two others species

Overall, the strategy employed for this work is ideal to solve suspected cases of cryptic diversity. The current DNA barcoding campaign for Lepidoptera offers a unique opportunity to facilitate taxonomic studies. With hundreds of thousands of specimens already barcoded in all families and with hundreds of specialists involved, these efforts are expected to greatly facilitate access to species identification (including larval stages) on one hand, and to provoke on the other hand an acceleration in the description of the large number of undescribed species in this insect order, in particular for the many overlooked cases of cryptic diversity for which morphology alone is insufficient. The DNA barcoding subcampaign for family Saturniidae already supported taxonomic work [8], [9] and a global release including most world species is in preparation (Rougerie et al., in prep.), benefiting from the participation of most expert taxonomists for the family.

Finally, we note that the phylogenetic analyses of the COI and 28S sequences failed to resolve the relationships between these three species; whereas COI sequences suggest that *S. malaisei* is sister to the pair formed by *S. bouyeri* and *S. myanmarensis* (Fig. 1A), this branching is poorly supported by our data (BS = 52). Although we could not polarize the subsitutions observed between the three species for the 28S dataset using a proper outgroup comparison, the presence of several shared indels in *S. bouyeri* and *S. malaisei* (Table 3) and the observation that these indels are absent from the only two homologous 28S sequences of Saturniidae present in GenBank (*Antheraea pernyi* HM359008, and *Samia ricini* AF463459 [erroneously placed in genus *Attacus* in GenBank]) suggest that the indels and many substitutions shared by *S. bouyeri* and *S. malaisei* may be derived characters which would then support *S. malaisei* and *S. bouyeri* as a pair of sister species (as in Fig. 1, B–C). In the absence of conclusive morphological characters to support one or the other hypothesis, or a better fitting biogeographical scenario, we consider however that the relationships between the three species are unresolved and deserve further study, as does the relationships between genus *Sinobirma* and its Madagascan and African relatives.

## Supporting Information

Figure S1 **Manual alignment of the 28S rRNA sequences for** ***Sinobirma myanmarensis*** **(barcode_snb_613 to 617),** ***S. malaisei*** **(BC-MNHN0016 to barcode_snb_1687) and** ***S. bouyeri*** **(BC-TB0167 to barcode_snb_1688).**
(PDF)

Figure S2 **Photograph of Kambaiti area (the current local spelling of the village is Kanpaiti) in September 2010 (photo SN).**
(TIF)
