# Supplementary figures and images for: Morphology and Molecules Reveal Unexpected Cryptic Diversity in the Enigmatic Genus Sinobirma Bryk, 1944 (Lepidoptera: Saturniidae)
Source: PLoS One. 2012 Sep 19;7(9):e43920. doi: 10.1371/journal.pone.0043920 (PMC3446977; doi:10.1371/journal.pone.0043920)

Fig. S11 - 28S rRNA sequence alignment

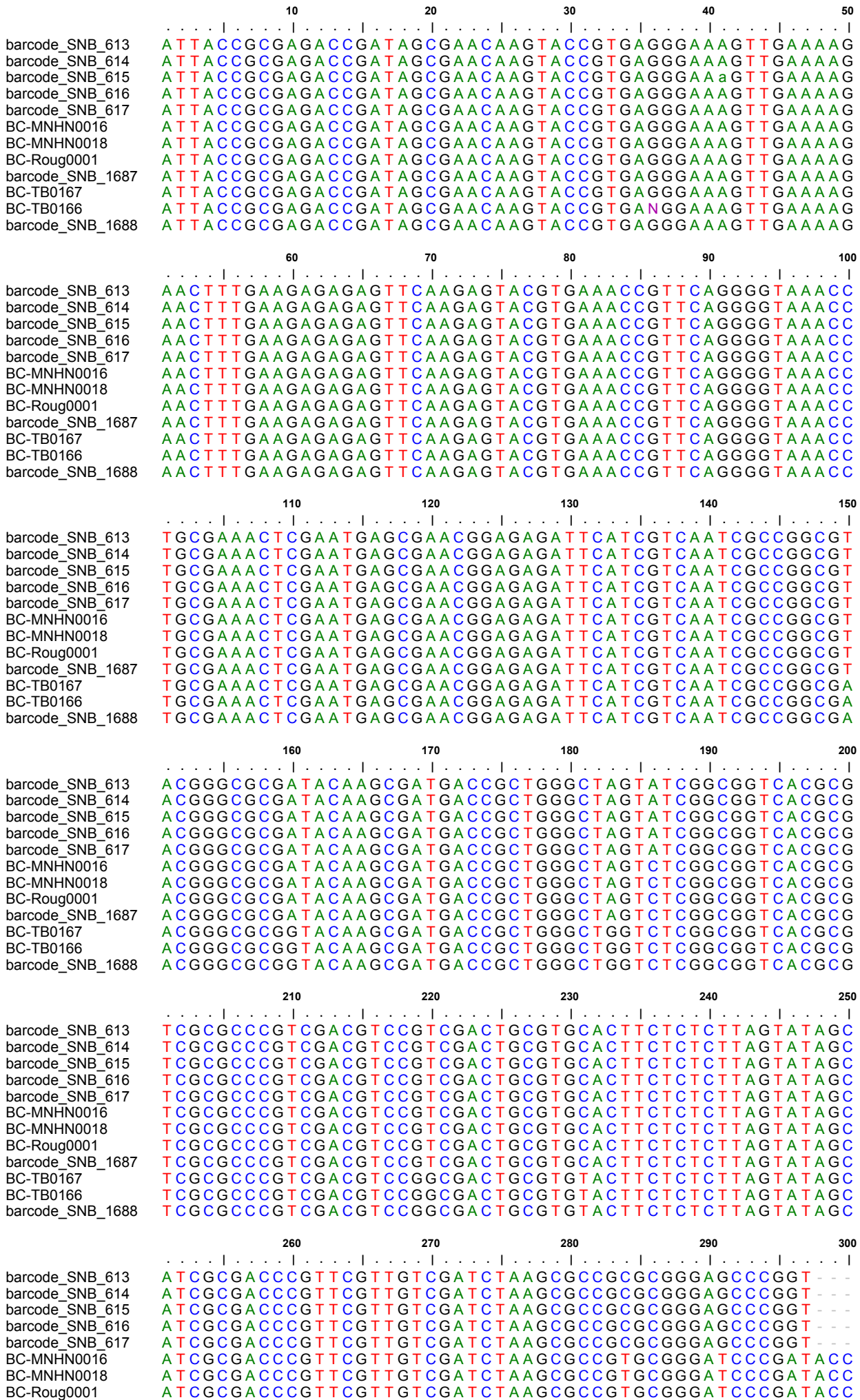

Fig. S11 - 28S rRNA sequence alignment

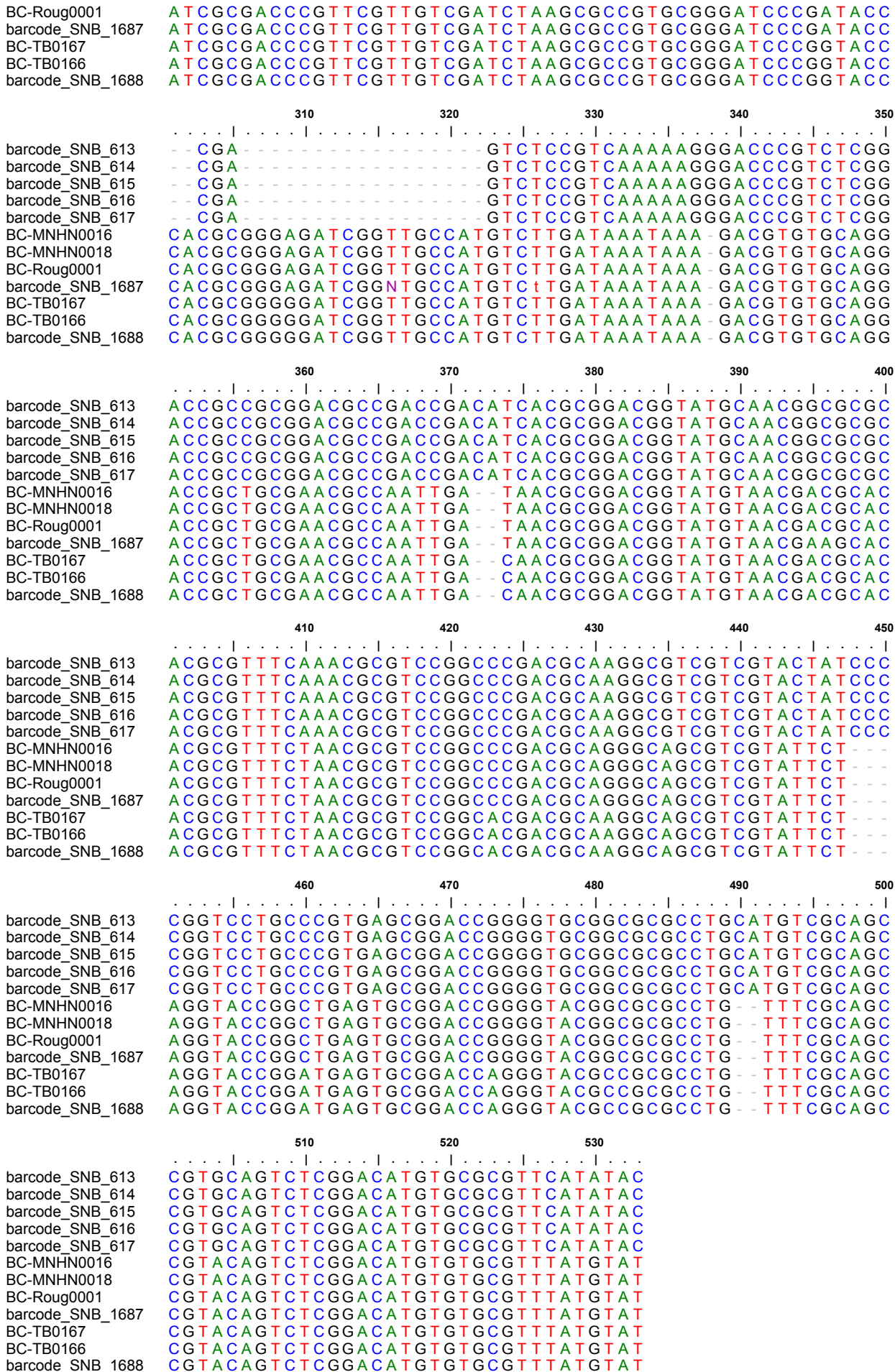

Supplement: Figure S1 — Manual alignment of the 28S rRNA sequences for Sinobirma myanmarensis (barcode_snb_613 to 617), S. malaisei (BC-MNHN0016 to barcode_snb_1687) and S. bouyeri (BC-TB0167 to barcode_snb_1688). (PDF) [file pone.0043920.s001.pdf]

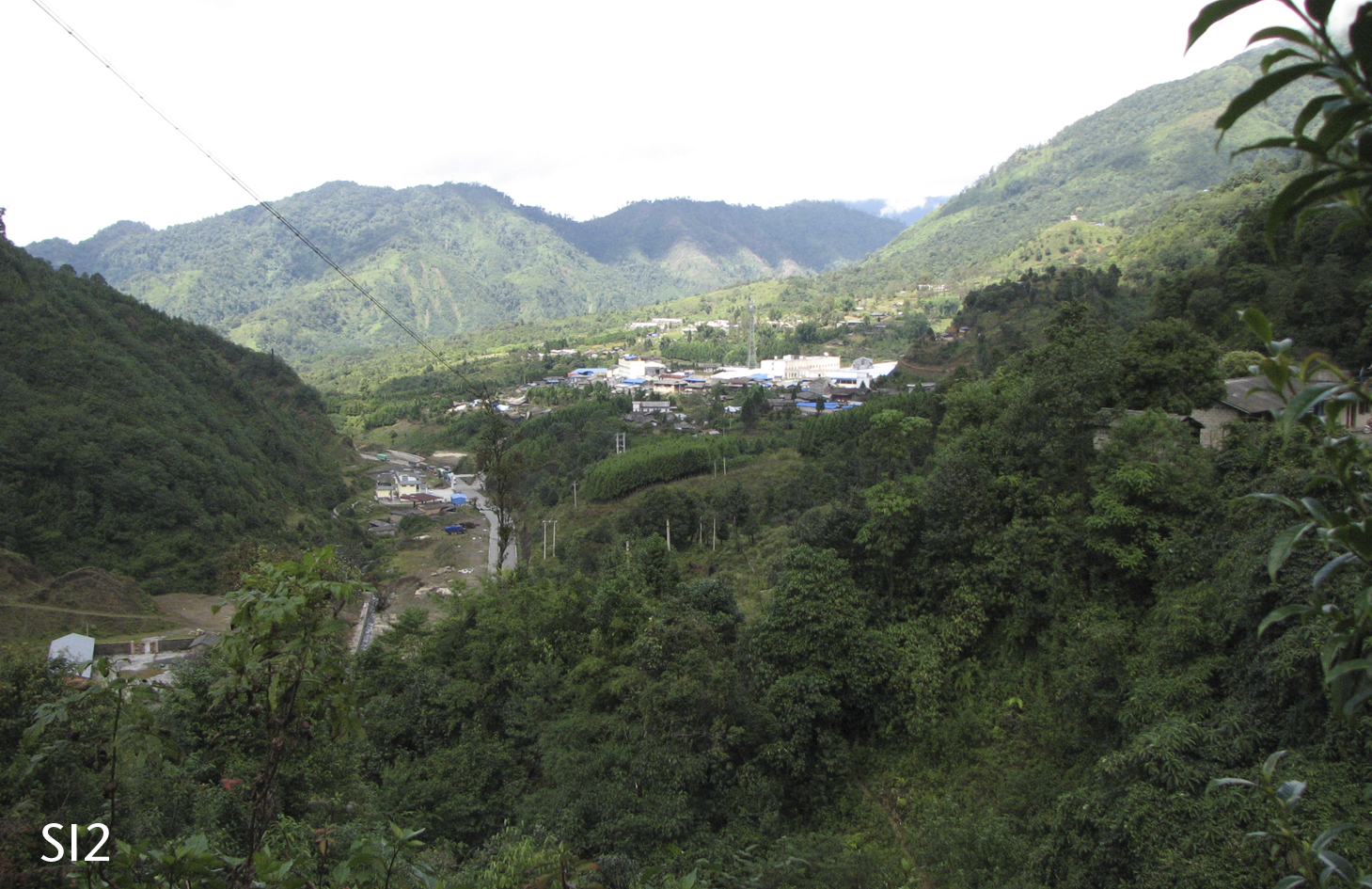

Supplement: Figure S2 — Photograph of Kambaiti area (the current local spelling of the village is Kanpaiti) in September 2010 (photo SN). (TIF) [file pone.0043920.s002.tif]
